# Enhancing Fingerprint Liveness Detection Accuracy Using Deep Learning: A Comprehensive Study and Novel Approach

**DOI:** 10.3390/jimaging9080158

**Published:** 2023-08-07

**Authors:** Deep Kothadiya, Chintan Bhatt, Dhruvil Soni, Kalpita Gadhe, Samir Patel, Alessandro Bruno, Pier Luigi Mazzeo

**Affiliations:** 1 U & P U Patel Department of Computer Engineering, CHA-RUSAT Campus, Charotar University of Science and Technology, Petlad 388421, India; deepkothadiya.ce@charusat.ac.in; 2 Department of Computer Science and Engineering, School of Technology, Pandit Deendayal Energy University, Gandhinagar 382007, India; 3 Department of Business, Economics, Law, Consumer Behaviour, IULM University, 20143 Milan, Italy; 4 ISASI Institute of Applied Sciences & Intelligent Systems-CNR, 73100 Lecce, Italy

**Keywords:** liveness detection, deep learning, computer vision, attention model, ResNet50

## Abstract

Liveness detection for fingerprint impressions plays a role in the meaningful prevention of any unauthorized activity or phishing attempt. The accessibility of unique individual identification has increased the popularity of biometrics. Deep learning with computer vision has proven remarkable results in image classification, detection, and many others. The proposed methodology relies on an attention model and ResNet convolutions. Spatial attention (SA) and channel attention (CA) models were used sequentially to enhance feature learning. A three-fold sequential attention model is used along with five convolution learning layers. The method’s performances have been tested across different pooling strategies, such as Max, Average, and Stochastic, over the LivDet-2021 dataset. Comparisons against different state-of-the-art variants of Convolutional Neural Networks, such as DenseNet121, VGG19, InceptionV3, and conventional ResNet50, have been carried out. In particular, tests have been aimed at assessing ResNet34 and ResNet50 models on feature extraction by further enhancing the sequential attention model. A Multilayer Perceptron (MLP) classifier used alongside a fully connected layer returns the ultimate prediction of the entire stack. Finally, the proposed method is also evaluated on feature extraction with and without attention models for ResNet and considering different pooling strategies.

## 1. Introduction

Over the last few decades, much scholarly attention has been paid to the weaknesses of biometric systems, which are victims of sensor attacks (conducted using synthetic biometric characteristics such as sticky fingers or high-quality printed photographs of the iris). In the digital age, automatic access to services is becoming increasingly crucial. As a result, the field of technology known as “biometric recognition” has emerged. 

In this regard, various standardization initiatives at the international level have been established to address security evaluation in biometric systems, such as the Common Standards through various Supporting Documents or the Biometric Evaluation Methodology. 

Subsequently, efforts in analyzing the direct attack weaknesses of automatic recognition systems have improved the security level provided by biometric systems. 

Various liveness detection techniques have been presented in the past few years. Anti-spoofing algorithms can differentiate between genuine and fake traits using multiple physiological properties. 

Due to their well-known uniqueness and persistence, fingerprints are one of the most significant biometric characteristics. In addition to being employed by forensic and law enforcement organizations worldwide, fingerprint recognition systems are also used in mobile devices for widespread use. 

Most fingerprint capture techniques need the finger to contact the capturing apparatus’s surface. Some sensor-based systems suffer from technical issues, such as low contrast from dirt or moisture on the plate of the capturing device or latent fingerprints from past users (ghost fingerprints). Hygiene issues reduce the attractiveness of contact-based fingerprint systems, which restricts their adoption, especially in multiuser applications. 

Priesnitz et al. [1] surveyed touchless fingerprint systems and compared them to traditional security measures based on something you know (PIN, password, etc.) or something you have; biometric technology offers several advantages (key, card, etc.) [2]. Conventional authentication methods cannot distinguish between imposters who have unlawfully obtained access rights and legitimate users and thus cannot substantiate false identity claims. 

Moreover, biometric systems do not require users to carry keys that may be misplaced or stolen or to memorize complicated PINs that are quickly forgotten. However, biometric facilities also have some drawbacks. Because external attacks on biometric systems could lower their level of security, it is essential to comprehend the risks they face. The vulnerability analysis plays the same importance in identifying potential attack points and suggests additional defences to increase their benefits to authentic users. Particular focus has been placed on direct attacks against the fingerprint recognition mechanism among the examined vulnerabilities. These attack techniques involve supplying a sensor with a fake fingerprint to identify the user as legitimate and give access [3].

The International Fingerprint Liveness Detection Competition (LivDet) is one of many projects highlighting the limitations and prospects of presentation attack detectors (PADs). The tournament developed seven editions between 2009 [4] and 2021 [5], presenting brand-new, complex falsification techniques, scanner types, and spoofs materials.

In this article, various software-based liveness detection systems are compared. To this end, several experiments have been carried out with several methods to estimate the top static and dynamic features for vitality identification and assess their effectiveness on the task. The same finger must be placed on the sensor and pulled off it more than once to obtain two or more static characteristics. Moreover, the dynamic features are taken from numerous image frames (i.e., several photos are captured once the finger is positioned on the sensor for a while) [6].

This work focuses on fingerprint liveness detection by investigating the effectiveness of ResNet50 architecture, attention principles, pooling strategies, and several classifiers on different datasets. 

Therefore, the contribution is threefold: (1) a proposed novel attention-based ResNet architecture for fingerprint liveness detection, (2) a thorough testing study to check the effectiveness of different pooling strategies, and (3) a comparative analysis with standard computer vision models and classifiers. 

The remainder of the article is organized as follows: Section 2 provides a literature survey and comparative analysis for liveness detection; Section 3 describes the proposed methodology to recognize the realness of finger impression; Section 4 deepens the description of the experiment; and Section 5 draws a line on discussions and conclusions.

### Literature Review

Using quality metrics to detect liveness has a new fingerprint parameterization that authors [7] have suggested. They have built the unique system and tested it on a development set of the LivDET competitions dataset, which comprises 4500 live and unreal images procured from three different kinds of sensors. The suggested method demonstrates robustness and accuracy in 93% of properly classified samples. Frassetto et al. [8] suggest using Local Binary Patterns and CNN with random weights. Both of these techniques are used in combination with Support Vector Machine (SVM). They have conducted several experiments on the dataset of the LivDET competition of the years 2009, 2011, and 2013 which comprised about 50,000 live and forgery fingerprint impressions taken from different sensors. When compared to previously reported findings, they reduced test error by 35%. Agarwal and A. Bansal [9] have proposed the fusion of pores perspiration and texture features in a static software-based approach. They carried out experiments on the LivDET 2013 and LivDET 2015 dataset, and their methods have also shown improvement in comparison to the state-of-the-art methods.

Tan [10] used 58 live, 80 spoof, and 25 cadaver participants for three separate scanners. They achieved an accuracy of around 90.9% to 100% using this particular dataset of spoof and live fingerprints. Sequeira et al. [11] introduced an automation-based image segmentation phase to separate the fingerprint impression from the backdrop. They have compared supervised learning approaches with image feature analysis methods. Dubey et al. [12] proposed to combine low-level gradient features with Speeded-Up Robust Features (SURF). They essentially extracted these features from a single fingerprint image to overcome the issues with dynamic software methodologies. Their results outperform the existing best average ERR by 9.625%.

Koshy et al. [13] designed a robust and multi-scenario dataset-based solution that achieves an accuracy of around 90% on classification. They only require one image from a finger to determine whether the input is live or phony. Ali et al. [14] analyzed SOTA (state-of-the-art) fingerprint recognition systems. They broke down the phases of fingerprint identification step by step and summarized fingerprint databases with relevant properties. The fingerprint liveness detection-based approach, based on DCNN and voting strategy, has been presented by Wang et al. [15], which outperforms handcrafted features methods and simultaneously optimizes feature extraction and classifier training.

Chowdhury et al. [16] worked with deep learning-based methods and models to benchmark them on the task. Eight additional scientific articles were compared, and they conducted their investigation using three distinct types of methodologies. 

Ahmad et al. [17] surveyed the scientific literature on the topic by looking at 146 crucial studies. Nur-A-Alam et al. [18] trained a multiclass classifier on specific properties has been proposed by the authors. They have performed various experiments that showed that the proposed methodology showed better performance with about 99.87% accuracy. Comparisons have also been conducted against recent machine learning classification techniques, such as SVM (97.86%) and Random Forest (95.47%).

Win et al. [19] targeted the field of criminal investigation by reviewing recent literature on fingerprint classification techniques and applications. They have also analyzed and compared the different computer vision and deep learning algorithms of finger impression images for classification. Priesnitz et al. [20] introduced a touchless fingerprint recognition-based methodology. They published approaches that range from self-identification fingerprint recognition to practice. At each stage of the recognition process, they additionally provide an overview of the state-of-the-art in the area of touchless 2D fingerprint recognition. Boero et al. [21] designed an Intrusion detection system (IDS) to analyze and detect security problems. The method focuses on anomaly detection and statistical analysis. They obtained the results which allow the best classifiers and show the performance that exploits the decisions of a bank of classifiers acting in parallel. Table 1 illustrates additional comparisons for reference.

## 2. Materials and Methods 

### 2.1. Methodology

**Figure 1:**
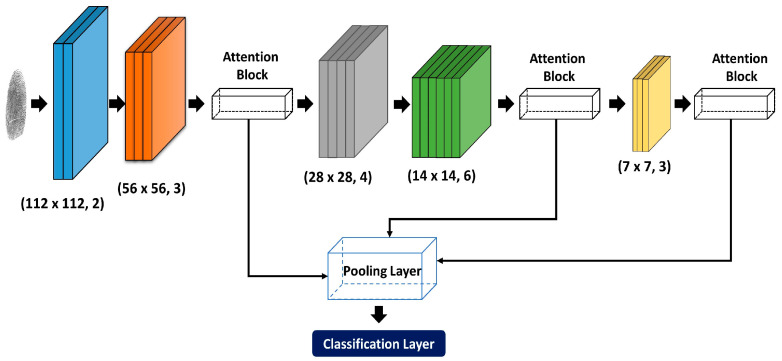
The architecture of the proposed attention-based convolution learning methodology.

The proposed methodology used ResNet50 architecture with an Attention mechanism to extract deep features from fingerprint images. Therefore, the proposed architecture is divided into three main modules (i) ResNet module, (ii) Pooling strategy, and (iii) Visual attention. Figure 1 shows the proposed methodology architecture consisting of five convolution ResNet blocks and three attention blocks featuring different pooling strategies.

### 2.2. Convolution Learning with ResNet50

**Figure 2:**
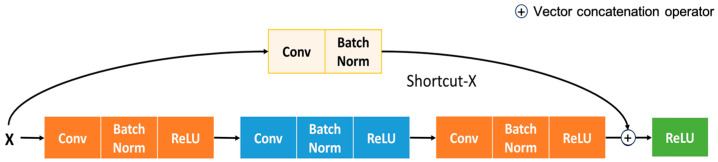
Functional architecture residual module with shortcut connection.

In this subsection, insights into Convolution Learning with ResNet50 are given. Vanilla ResNet architecture has been used for convolution learning, with skip connection to counter overfitting and gradient explosion. The residual module aims to add the features extracted from the neural networks. On top of that, short connections are used to reduce the vanishing gradient problem. Figure 2 represents the internal structure of convolution learning with ResNet as a backbone. The introduction of batch normalization allows for increasing convolution speed and stability. Equation (1) shows the normalization process adopted in ReLU’s activation to normalize features.

(1) $$f\left(x\right)=\{\begin{array}{l} x,\;if\;x>0\\ 0,\;otherwise \end{array}$$

### 2.3. Visual Attention 

**Figure 3:**
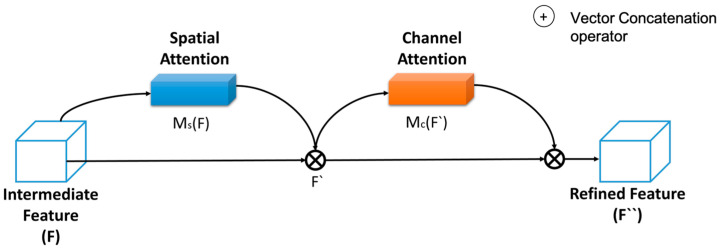
Proposed channel attention and spatial attention sequential model.

The proposed methodology relies on attention models to enhance feature learning capabilities and prevent the loss of features caused by single pooling. The proposed method adopts spatial- and channel-based sequential attention modules. In particular, three sequential attention modules represent the backbone network of ResNet. Figure 3 provides a graphical depiction of the functional structure of the spatial-channel attention block.

**Figure 4:**
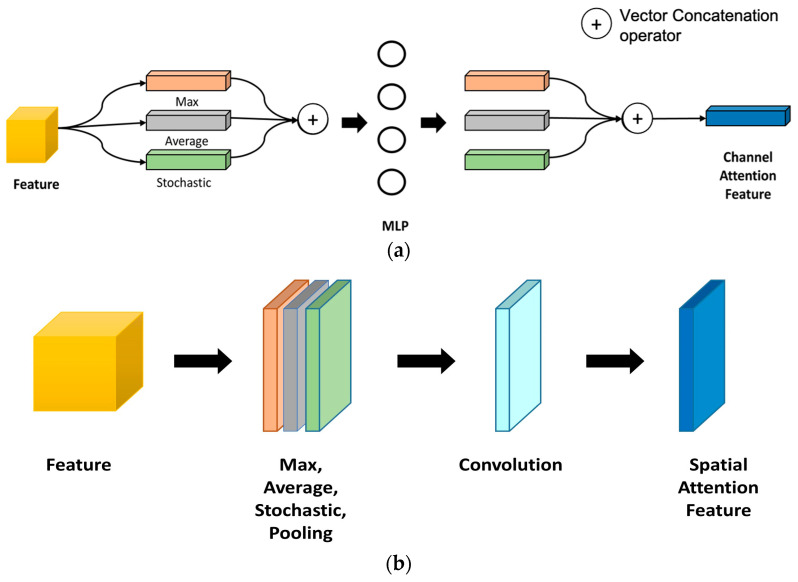
(**a**) Functional structure of proposed channel attention block; (**b**) Functional structure of proposed spatial attention block.

The concatenation of basic channel attention modules (CA) and spatial attention modules (SA) relies on mixed attention [26]. The internal structure of the CA and SA blocks are displayed in Figure 4a and Figure 4b, respectively.

CA module allows to compute the relevant links between distinct channels. In addition, the SA block accounts for executing multiple types of visual attention to obtain information on content, texture, and background. 

Feature Map F(C × H × W) is passed through the global average pooling to generate $F_{c}\left(1\times 1\times C\right)$, for C channels. Then, it is forwarded to MLP (Multilayer Perceptron) with four (4) hidden layers. The activation of the hidden layer was set to scale the output as $R^{\left(1\times 1\times \frac{C}{r}\right)}$ to adjust the ResNet map according to the compression rate. 

The final map $M_{c}\left(F\right)$ is computed as in Equation (2), where *W* and *b* are weights and biases for channel attention $R^{c}$.

(2) $$M_{c}\left(F\right)=Norm\left(W_{1}\left(W_{0}\;AvgPool\left(F_{c}\right)+b_{0}\right)+b_{1}\right)$$

SA generates intermediate spatial features Fs(H × W × 1) to empower spatial locations. Input feature $F_{c}$ adopts full 3 × 3 convolution to extract the information from the input matrix. A higher spatial attention dimension adopts $R^{\left(H\times W\times 1\right)}$ through 3 × 3 convolution. 

Finally, extracted SA output $M_{s}\left(F\right)$ is generated with Batch normalization as in Equation (3).

(3) $$M_{s}\left(F\right)=Norm\left(AvgPool\left(f_{0}^{3\times 3}\left(F_{c}\right)\right)\right)$$

Here, Equation (3) is generalized with global average pooling.

In addition, the simulation of the model also explores different pooling strategies to find the most appropriate based on input image property. Element-wise multiplication is used to produce refined intermediate features from the attention blocks. They are represented as spatial attention features F(s) and channel attention features Fc and used in Equations (4) and (5) to calculate the final output of the attention block $F^{\prime \prime }$ as an intermediate feature, while F is considered an input feature for the attention block.

(4) $$F^{\prime }=M_{c}\left(F\right)\;⊗\;F$$

(5) $$F^{\prime \prime }=M_{s\;}⊗\;F\prime$$

### 2.4. Pooling Operation

**Figure 5:**
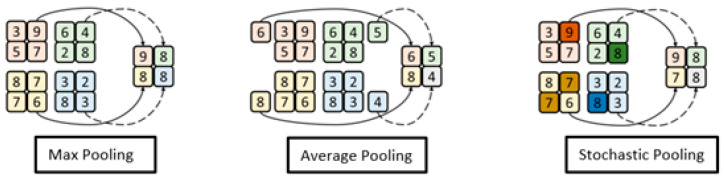
Various pooling strategies employed in the proposed architecture.

The proposed methodology also tests three pooling strategies to optimize performance over fingerprint liveness detection. That is motivated by pooling method performances being dependent on the nature of targeted images. Pooling plays a critical role in convolution learning in extracting information from learned features. It also brings in a dimension reduction. CNN-based architecture has common pooling strategy as max pooling, average pooling, and stochastic pooling, as illustrated in Figure 5. The pooling strategy can be selected based on image background, channel type, texture, and many more features. For instance, non-maximum values in the pooling kernel are discarded by max pooling, while the maximal feature values are not retained by average pooling. Conversely, retention of features in a certain direction is not the primary objective of stochastic pooling. Therefore, sticking to one strategy of pooling may lead to limited classification performance. 

### 2.5. Algorithm 

The computational features that have been previously described are processed by a three-layer MLP [27] network to detect the liveness of fingerprints. For the sake of clarity, a description of the training procedure for the Dual Attention model is provided in the form of Algorithm 1 down below. The scripting structure of the proposed dual attention-based methodology demonstrates the major functional component of the architecture.
**Algorithm 1:** Training procedure for Dual Attention modelOutput: Attention features *F*
 Pol(x) ← P{Max, Avg, Stoch} Mc ← Channel Attention Model Ms ← Spatial Attention Model model ← ResNet():       model.FcLayer ← ReLU()       mode.Attention ← f (Mc ⊗ Ms)       model.Pooling ← $f\left(Pol_{x}\right)$ for epoch =0 to epoch:       for (x_train, y_train):            x ← model.normalization (x_train, kernel)            y ← model.Conv( $\sum^{\;}f\left(Weight_{x}\;⊗\;Input_{x}\right)$)            a ← model.attention(pol(y) =             z ← model.Pooling( $\overset{2}{\underset{0}{\sum }}f\left(Pol\_a_{x}\right)$)             α. ← model.FcLayer (MLP(z))            *f*(*x*) ← optimization.adam(α)            *loss* ← loss.$\overset{n}{\underset{i}{\sum }}y_{i}\;\log\left(\alpha_{i}\right)$
       end for end for        Return: *f*(*x*), *Feature of training dataset*

## 3. Results

### 3.1. Dataset

The proposed methodology has been tested over two different datasets: LivDet DB [28] and ATVS DB [29]. The former consists of three different types of images: (i) Biometrika, (ii) CrossMatch, and (iii) Identix. Fack fingerprints are designed with silicone, gelatine, and playdoh. ATVS DB consists of different datasets having three different techniques: (i) flat optical Biometrika, (ii) flat capacitive Precise, and (iii) thermal sweeping Yubee (demonstrated in Table 2). 

### 3.2. Data Augmentation

**Figure 6:**
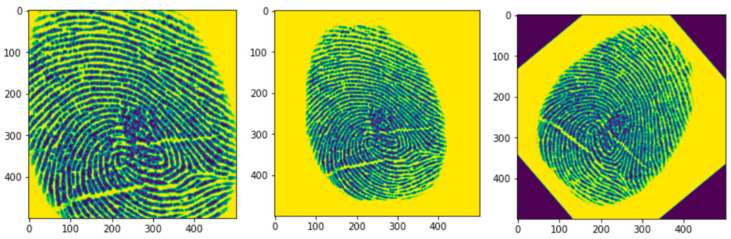
Manual Augmented dataset used in the proposed methodology.

The proposed methodology relies on data augmentation for targeted datasets to increase the knowledge inference capabilities of the model. Generally, data augmentation methods include rotation, scaling, flip, crop, and many more. Among them, angular rotation (±20°) and scaling were used to increase the training dataset. Figure 6 represents some augmented samples from the training dataset.

### 3.3. Experiments and Result

This section provides the reader with the experimental settings and results. Several trials have been carried out with ResNet architecture with different depths: ResNet50 and ResNet34. Dual attention blocks were embedded at a different level of convolution. As shown In Table 3, the model stack includes convolution layers, SA, CA, pooling, and activation functions. It features an initial kernel size set to 7 × 7, and the final fully connected layer is characterized by ReLU activation. 

The training phase has been carried out with the following parameters and settings: 20 epochs; Adam optimizer; learning rate set to 10 to the power (-3); binary cross-entropy as loss function and accuracy metrics to evaluate performances; batch size set to 16; dropout equals to 0.3. The training and testing tasks have been run on an NVIDIA GeForce RTX 3080 (AIDA Lab KSA, Riyadh Saudi Arabia) and are coded with Python 3.10. 

The proposed dual attention-based methodology has achieved a 97.78% accuracy rate over the LivDet dataset. The proposed model was evaluated using Sensitivity [30], Precision [31], and F1-score [32], mathematically described in Equations (6)–(8).

(6) $$\mathrm{Sensitivity}=\frac{\mathrm{TP}}{TP+FN}\;$$

(7) $$\mathrm{Precision}=\frac{\mathrm{TP}}{TP+FP}$$

(8) $$F1-\mathrm{score}=\frac{2*\mathrm{TP}}{2*TP+FP+FN}$$

Table 4 represents a statistical evaluation of the proposed methodology with ResNet34 and ResNet50 on two different datasets (LivDet and ATVS). 

**Figure 7:**
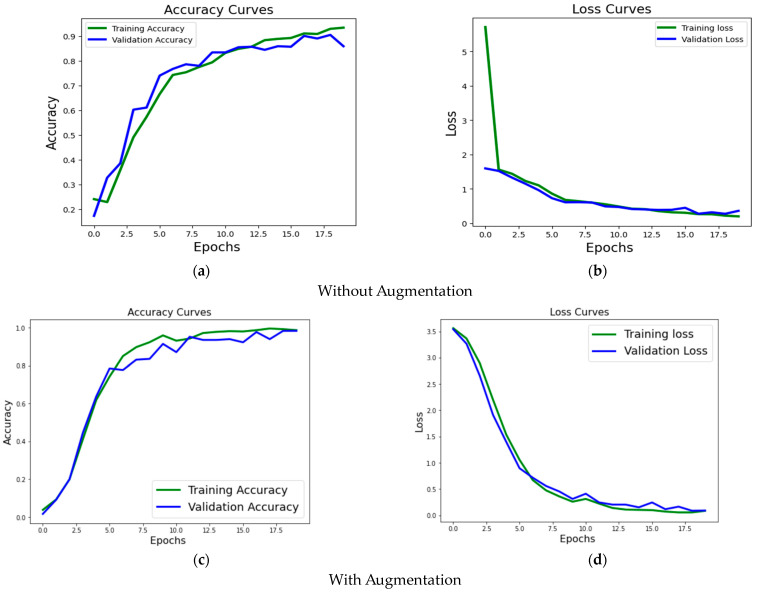
Accuracy and loss evaluation of the proposed methodology over the LivDet dataset without augmentation are given in subfigures (**a**,**b**). The same metrics are graphically depicted in subfigures (**c**,**d**) for the experiments with augmentation.

The accuracy metrics and loss function (Figure 7) allow benchmarking training and validation steps for the proposed method. In particular, Figure 7a,b illustrate the results of the proposed methodology for the ResNet50 model without augmentation. At the same time, subplots (c) and (d) draw the corresponding curves for the training with augmented data. 

Table 5 shows ResNet50 and ResNet34 architectures are compared with and without attention modules. Experiments have been run using three different pooling strategies (Table 6). Stochastic pooling offers remarkable accuracy over average and max pooling for the proposed Res-Net50-based methodology on the LivDet dataset. 

However, the selection of the pooling strategy highly depends on the input type. 

**Figure 8:**
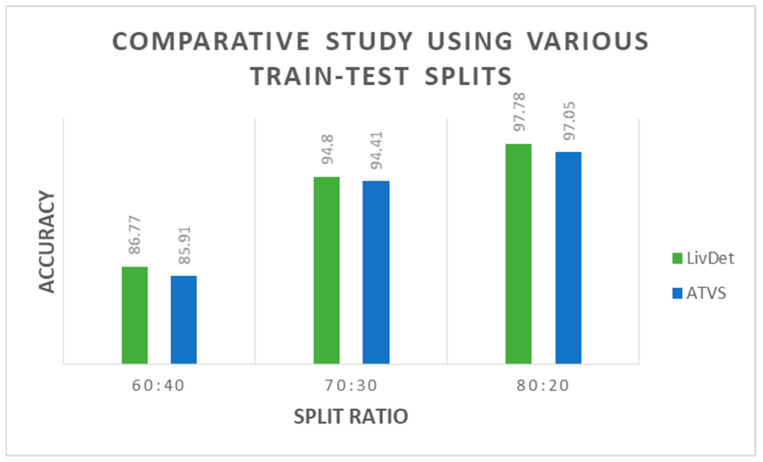
Comparison study considering various train-test ratios for the proposed architecture.

Table 7 shows a comparative analysis of ResNet50 with different learning rates and Dropout values on both datasets. The experiments reveal a 0.0001 Learning Rate and a dropout of 0.3, being the combination performing higher. Some other tests have been run with various train test split ratios, as shown in Figure 8. 

**Figure 9:**
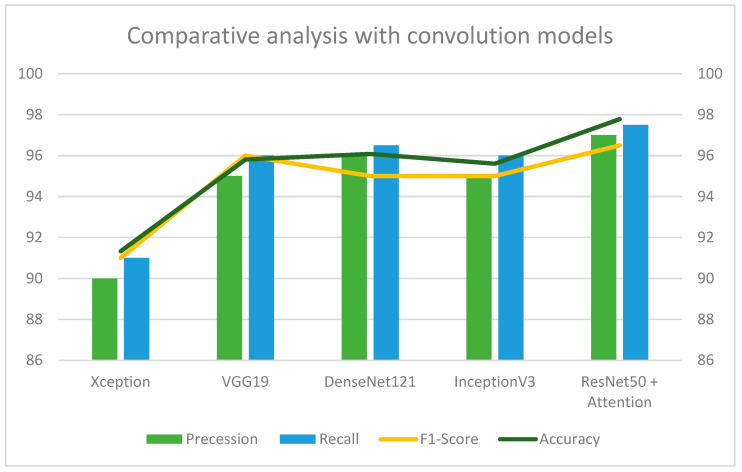
The proposed methodology has been compared against other deep learning models on the LivDet dataset.

Figure 9 represents a comparative analysis of the proposed sequential attention model against other state-of-the-art convolution models such as VGG19, Densenet121, and InceptionV3, and different optimizers such as Gradient Descent, Stochastic Gradient Decent (SDG), and Adam [33]. Table 8 shows a comparative analysis with different optimizers and different deep learning models. 

## 4. Discussion

The proposed study adopts an attention-based technique to detect liveness from fingerprint images and recognize them as real or fake. The proposed methodology uses a unique approach to see the realness of figure print from pictures as dual attention-based Resnet50 architecture has achieved 97.78% remarkable accuracy on the LivDet fingerprint dataset. Experiments have been carried out on ATVS for a more extensive analysis of the proposed technique. 

**Figure 10:**
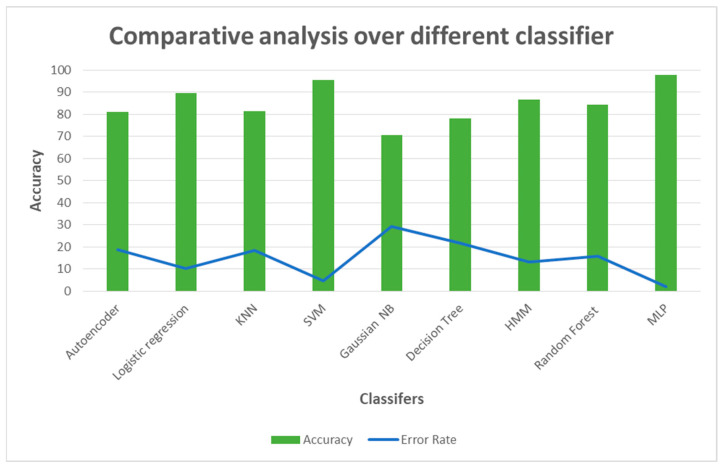
Comparative analysis of the proposed dual attention-based model with different classifiers.

Furthermore, the proposed methodology evaluates the robustness of different pooling strategies to extend accuracy or avail results. In particular, the experimental campaign in this work reveals the stochastic pooling method outperforming Max and Average pooling on the fingerprint recognition task. The architecture proposed heavily relies on attention blocks: spatial and channel attention modules are employed to enhance feature extraction. The proposed methodology is superior to other deep learning convolution models, such as Xception, InceptionV3, VGG19, DenseNet121, and InceptionV3, with around 0.7% accuracy. Further investigations have been conducted concerning the employment of classifiers (Figure 10), such as Random Forest (RF) [34], Linear Regression (LR) [35], KNN [36], SVM [37], Gaussian NB [37], Decision Tree [38], HMM [39], Autoencoder [40] and Support Vector Machine (SVM) [41]. Convolution layer five output from ResNet50 is converted into a 2048-dimensional array, which feeds all comparison classifiers. In that case, the ResNet50 stack is used until convolutional layer five only for feature extraction, represented by the 2048-dimensional array. 

As noticed in Figure 10, Multilayer Perceptron (MLP) has proven higher accuracy than other machine learning classifiers. 

**Figure 11:**
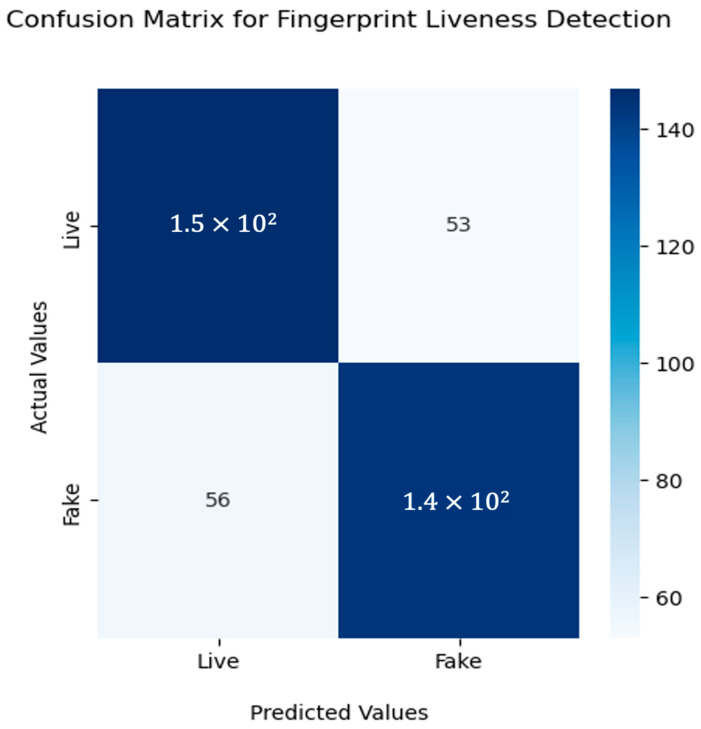
Confusion matrix for fingerprint liveness detection using Resnet50 with sequential attention model.

Figure 11 illustrates the confusion matrix of the proposed methodology on the LiveDet dataset, with 53 images misclassified as fake out of 1500 images. 

The proposed dual attention-based ResNet50 accounts for 50 layers in the neural network, bringing in not negligible computational costs.

## 5. Conclusions

The digital fingerprint is a powerful applicable tool for a plethora of scenarios, such as person authentication in commercial business, civil and forensic usage, etc. Most fingerprint-based scanners proved robust and accurate in fingerprint recognition. They stand almost 100% accuracy during authentication. 

In this study, a vision-based methodology has been introduced to focus on fingerprint liveness detection. As previously mentioned, the contribution of this work is threefold: (1) the introduction of a novel attention-based ResNet architecture for fingerprint liveness detection, (2) a thorough testing study to check the effectiveness of different pooling strategies, (3) a comparative analysis with standard computer vision models and classifiers.

An attention-based learning approach to recognize the liveness of fingerprint images is proposed to tackle fingerprint liveness detection. To this end, the methodology relies on ResNet as the architecture backbone for convolution learning. In particular, two attention module channels followed by spatial attention are featured in the architecture. The architecture has been tested on three different pooling strategies with, interestingly, some positive outcomes achieved with Stochastic pooling. 

Furthermore, the extensive experimental campaign conducted over two different datasets showed the positive impact of dual attention-based learning on the correctness of the results. Comparisons to other machine learning and deep learning models, such as Random Forest and CNN variants, such as VGG19 and DenseNet121, also confirm that. 

Feature work for this study to enhance the model toward more accuracy and robustness for real-time scenarios. The proposed methodology is meant to be extended to detect presentation attacks using other biometrics, such as retina and face. The proposed study is also planned to be improved by integrating Explainable AI to interpret the prediction of the proposed deep learning model. 

## Figures and Tables

**Table 1:** Comparative analysis of fingerprint presentation attack detection using different deep learning models.

Author	Year	Approach	Result
Y Zhang et al. [22]	2020	FLDNet	91% Acc
Y Zhang et al. [23]	2019	JLW_B	95.25 Acc
C Yuan et al. [24]	2020	DCNN	95.35 Acc
Z Xia et al. [25]	2018	FLD + SVM	5.69 CE

**Table 2:** Describes the characteristics of the targeted dataset.

Dataset	Class	Real/Live	Fake	Avg. Resolution
LivDet [28]	2	5000	3000	580 dpi
ATVS [29]	2	4800	4000	520 dpi

**Table 3:** ResNet34 and ResNet50 network stacks and the corresponding Channel attention (CA) and Spatial attention (SA) modules are described below.

Conv-Layer	Output Size	ResNet34	RetNet50
01	112 × 112	7 × 7, 64	7 × 7, 64
02	56 × 56	$\left[\begin{array}{c} 3\times 3,\;64\\ 3\times 3,\;64 \end{array}\right]$ × 3	$\left[\begin{array}{c} \begin{array}{c} 1\times 1,\;64\\ 3\times 3,\;64 \end{array}\\ 1\times 1,\;256 \end{array}\right]\times 3$
	Attention (CA + SA)		
03	28 × 28	$\left[\begin{array}{c} 3\times 3,\;128\\ 3\times 3,\;128 \end{array}\right]$ × 4	$\left[\begin{array}{c} \begin{array}{c} 1\times 1,\;128\\ 3\times 3,\;128 \end{array}\\ 1\times 1,\;512 \end{array}\right]\times 4$
04	14 × 14	$\left[\begin{array}{c} 3\times 3,\;256\\ 3\times 3,\;256 \end{array}\right]$ × 6	$\left[\begin{array}{c} \begin{array}{c} 1\times 1,\;256\\ 3\times 3,\;256 \end{array}\\ 1\times 1,\;1024 \end{array}\right]\times 6$
	Attention (CA + SA)		
05	7 × 7	$\left[\begin{array}{c} 3\times 3,\;512\\ 3\times 3,\;512 \end{array}\right]$ × 3	$\left[\begin{array}{c} \begin{array}{c} 1\times 1,\;512\\ 3\times 3,\;512 \end{array}\\ 1\times 1,\;2048 \end{array}\right]\times 3$
	Attention (CA + SA)		
Fully Connected	1 × 1	Pooling, Activation	

**Table 4:** Comparative analysis of ReNet34 and ResNet50 with attention modules over both datasets are given below.

Model	Dataset	Sensitivity	Precision	F1-Score	Accuracy
ResNet34 + Attention	LivDet	0.94	0.95	0.95	95.81%
	ATVS	0.95	0.95	0.95	95.52%
ResNet50 + Attention	LivDet	0.97	0.97	0.97	97.78%
	ATVS	0.96	0.97	0.96	97.05%

**Table 5:** Comparative analyses of ResNet variants with and without attention models for the LivDet dataset are given below.

Model	Attention	Avg. Precision	Avg. Recall	Avg. F1-Score
ResNet34	Yes	0.95	0.96	0.95
	No	0.83	0.84	0.84
ResNet50	Yes	0.97	0.97	0.97
	No	0.87	0.86	0.86

**Table 6:** Comparative analysis is given for different pooling strategies for the Attention-based ResNet50 model on the LivDet dataset.

Database	Max Pooling	Average pooling	Stochastic Pooling
LivDet	97.23%	97.14%	97.78%
ATVS	96.00%	96.33	97.05%

**Table 7:** Accuracy analysis with a combination of different Learning rates and dropout rates for proposed architecture with ResNet50.

Learning Rate	0.01			0.001			0.0001		
Dropout	0.1	0.2	0.3	0.1	0.2	0.3	0.1	0.2	0.3
LivDet	90.03	94.5	95.22	94.23	96.61	97.33	96.86	96.96	97.78
ATVS	88.64	92.74	94.12	93.88	96.35	96.98	96.41	96.52	97.05

**Table 8:** Comparative analysis with different optimizers and models trained over both the scenarios, with and without association of attention model for ResNet34 and ResNet50.

Model	Optimizer	Accuracy	Precision	Recall
ResNet34	GD	77.46%	0.79	0.75
	SGD	82.60%	0.83	0.83
	Adam	83.56%	0.83	0.84
ResNet34 + attention	GD	85.30%	0.88	0.85
	SGD	94.21%	0.94	0.94
	Adam	95.81%	0.95	0.96
Resnet50	GD	71.05%	0.71	0.72
	SGD	84.71%	0.85	0.85
	Adam	87.21%	0.87	0.86
Resnet50 + Attention	GD	88.49%	0.88	0.87
	SGD	95.90%	0.96	0.97
	Adam	97.78%	0.97	0.97

## Data Availability

Data are available from the LivDet 2021 challenge upon reasonable request.
